# Prevalence, infection intensity and geographical distribution of schistosomiasis among pre-school and school aged children in villages surrounding Lake Nyasa, Tanzania

**DOI:** 10.1038/s41598-020-80317-x

**Published:** 2021-01-11

**Authors:** Humphrey Deogratias Mazigo, Cecilia Uisso, Paul Kazyoba, Andreas Nshala, Upendo J. Mwingira

**Affiliations:** 1 Department of Medical Parasitology, School of Medicine, Catholic University of Health and Allied Sciences, P.O. Box 1464, Mwanza, Tanzania; 2 National Neglected Tropical Diseases Control Programme, National Institute for Medical Research, 3 Barack Obama Drive, P.O. Box 9653, 11101 Dar-Es-Salaam, Tanzania; 3 National Institute for Medical Research, 3 Barack Obama Drive, P.O. Box 9653, 11101 Dar-Es-Salaam, Tanzania; 4 RTI International, 701 13th Street NW, Washington DC, 20005 USA

**Keywords:** Diseases, Infectious diseases

## Abstract

Planning and implementation of schistosomiasis control activities requires an understanding of the prevalence, intensity of infection and geographical distribution of the disease in different epidemiological settings. Although, Tanzania is known to be highly endemic to schistosomiasis, there is paucity of data on the geographical distribution of schistosomiasis in potential large water bodies in the country. Thus, the present study was conducted to determine the prevalence, infection intensities and geographical distribution of schistosomiasis along villages located on the shoreline of Lake Nyasa, southern Tanzania. A cross-sectional study was conducted among 1560 children aged 1–13 years old living in villages located along the shoreline of Lake Nyasa. A single urine and stool sample was obtained from each participating child and screened for *S.* *mansoni* using Kato Katz (KK) technique to detect eggs and using point-of-care circulating Cathodic Antigen (POC-CCA) test to detect antigen in urine. Urine filtration technique was used to screen for *S.* *haematobium* eggs in urine samples. Villages/primary school were mapped using geographical information system and prevalence map was generated using ArcView GIS software. The overall prevalence of *S.* *mansoni* based on KK technique and POC-CCA test was 15.1% (95%CI: 13.4–16.9) and 21.8% (95%CI: 18.5–25.3) respectively. The prevalence *S.* *haematobium* was 0.83% (95%CI: 0.5–1.4) and that of haematuria was 0.9%. The arithmetic mean egg intensities for *S.* *haematobium* and *S.* *mansoni* were 18.5 mean eggs/10 ml (95%CI: 5.9–57.6) of urine and 34.7 mean epg (95%CI: 27.7–41.7) respectively. Villages located on the southern end of the lake had significantly high prevalence of *S.* *mansoni* than those located on the northern part (χ^2^ = 178.7838, *P* = 0.001). Cases of *S.* *haematobium* were detected only in three villages. Both *S.* *mansoni* and *S.* *haematobium* infections occur in villages located along the shoreline of Lake Nyasa at varying prevalence. These finding provide insights that can provide guidance in planning and implementation of MDA approach and other recommended measures such as improvement in sanitation, provision of clean water and behaviour changes through public health education.

## Introduction

The sub-Saharan African region, carries 90% of the over 250 million cases of schistosomiasis occurring worldwide^1,2^. In this region, after Nigeria, Tanzania is second country having the highest cases of schistosomiasis and approximately 51.5%0 of the Tanzanian population is either exposed or live in areas with high risk of exposure^3,4^. The country is endemic to both *Schistosoma* *mansoni* and *Schistosoma* *haematobium*, these infections are common in communities characterised with limited access to water, sanitation, hygienic practices and health services^4^. *Schistosoma* *mansoni* infection is associated with hepatosplenic disease characterised with hepatomegaly, splenomegaly, progressive periportal fibrosis (PPF) which can lead to portal hypertension and its related sequelae, mainly ascites, liver surface irregularities, oesophageal varices and haematemesis^5–7^. The main consequences of *S.* *haematobium* infection are haematuria, dysuria, nutritional deficiencies, urinary bladder lesions, hydronephrosis, urinary bladder squamous cell carcinoma and in children, growth retardation^8^. Preventive chemotherapy using mass drug administration (MDA) of praziquantel targeting primary school aged children is the main strategy for controlling schistosomiasis in Tanzania^9^.

An important aspect for establishing preventive chemotherapy strategy for schistosomiasis is to understand the geographical distribution of the disease and the infection level in endemic communities living in different geographical settings^10–13^. It remains important to identify areas where infections have continued to be a public health problem despite repeated rounds of MDA, this will allow development of a focused integrated control measures^14,15^. In many of the schistosomiasis endemic countries, this has never been the case, for example in Tanzania, there is inadequate attention given to research on the geographical distribution of schistosomiasis in other areas outside the historically known and highly researched areas^16–19^. This paucity of data affects the designing, implementation, monitoring and evaluation of control interventions. In Tanzania, the general knowledge indicate that transmission of schistosomiasis occurs within large freshwater water bodies such as lakes, dams, irrigation schemes and in seasonal/temporal water bodies^4^. The north-western region located along the Lake Victoria basin and the southern part of the Lake Victoria are known to be highly endemic to both *S.* *mansoni* and *S.* *haematobium* infections^4^. However, there is limited information on the geographical distribution of schistosomiasis in the two other large water bodies in Tanzania, Lake Tanganyika in the western region^4,20,21^ and Lake Nyasa in the southern highlands^4,22^. We focused on the Lake Nyasa, in the southern part of the country, on the international border between Malawi, Mozambique and Tanzania. Available information from Malawi, indicate that villages surrounding Lake Malawi are highly endemic to *S.* *haematobium* with the prevalence ranging from 21 to 72.7%^23^, whereas inland villages have moderate prevalence ranging from 10.2 to 26.4%^23^. The prevalence of *S.* *mansoni* infection is low in the lakeshore communities (3.9%)^23^. In contrast, the endemicity levels of schistosomiasis disease/infection in villages surrounding Lake Nyasa on the Tanzanian side is not known. The lack of epidemiological data on the Tanzanian side of the lake may present a unique challenge for the control efforts on each side of the border/lake. In addition, this has implication in the design and implementation of control measures especially MDA at this time when resources are very scarce and without data on geographical distribution of schistosomiasis, resources might be allocated to areas where they are not needed or underestimated^24,25^. Thus, accurate and up-to-date information on schistosomiasis infection/disease is essential for informing the design and implementation of a cost-effective control interventions and monitoring the impact of MDA at village, district, regional and national levels^24^. In addition, describing the geographical distribution and prevalence of infection in areas bordering countries opens cross-border cooperation and collaboration to fight against neglected tropical diseases^23^. The mapping exercises always target the most at risk population such as school aged children to demonstrate the true prevalence and burden of the infection^16^. Several studies have demonstrated that school aged children harbour the highest prevalence and intensity of schistosome infections^1^. Recent data shows that pre-school aged children are also infected with schistosomes at early ages and carries heavy intensity of infection^26^. Thus, inclusion of the highly risk two groups in the mapping surveys will demonstrate a clear picture of the infection and transmission status of the disease. In that context, the present cross-sectional study was conducted to determine the prevalence, infection intensities and geographical distribution of schistosomiasis (both *S.* *haematobium* and *S.* *mansoni*) infection among pre-school aged children and school aged children living in villages surrounding Lake Nyasa, in Nyasa District, Southern Tanzania. Understanding the prevalence and geographical distribution not only help in identifying high risk communities but also help in planning and implementation of mass drug administration based on level of infection^24^.

## Methods

### Study area

Nyasa district is among the six (6) districts of Ruvuma region and the district lies between latitudes 10,015′N and 1103′S and longitudes 34,024′W and 350,228′E. The district has a total area of 3811 km^2^ and located between 1200 and 2000 M above the se level. The district, border Lake Nyasa to the West. Lake Nyasa/lake Malawi is located on the southern part of the East African Rift valley and is an international border of Malawi, Mozambique and Tanzania. The districts border Mozambique to the south and Malawi to the West (separated by Lake Nyasa). According to the 2012 national census, the district was occupied by 146,160 inhabitants^27^.

In terms of topography, the Livingstone mountains forms the narrow stretches of lowland to Lake Nyasa and the mountains ranges are the main sources of seasonal rivers and streams flowing down the Lake. The district is characterised by uni-modal rainfall pattern starting from November or December and ends in April/May. On average, the districts receive an approximately 1224 mm of rainfall per year and temperature ranges from 29 to 31 °C and drops between 19 and 23 °C during June and August (the cold season). The main economic activities of the inhabitants are mainly subsistence farming growing cassava, maize and sweet potatoes and fishing. Fishing is the main economic activity practiced in villages located along the shorelines of the Lake Nyasa. The study was conducted in villages located within a radius of 5-km from the shoreline of Lake Nyasa basin and they included Hongi, Chiulu, Kwambe, Litimba, Liwundi, Lundo, Lundu, Mbuli, Tumbi and Mbaba-bay. Schistosomiasis control in the district mainly focus on MDA targeting primary school aged children and is organized and implemented by the district health department under the National Neglected Tropical Diseases Control Programme. No any other schistosomiasis intervention measure are implemented in the district under the district NTD control programme.

### Study design, inclusion and exclusion criteria

This was a cross-sectional study conducted from September to October, 2019 among pre-school (aged 1–5 years) and school aged children (aged 6–13 years from class 1–5) in selected villages surrounding Lake Nyasa. Based on previous knowledge among school children of inverse relationship between prevalence of *S.mansoni* and proximity to the lake^10,25^ and the knowledge that the prevalence of *S.haematobium* increases with increase in distance to inland from the lake shore in north-western Tanzania^4^, village located within a radius of 5 km within the lake basin were selected for the study. The study included children aged 1–13 years, living in the study villages, having no history of participating in previous rounds of MDA in the past 6 months prior to the study, who were either male or female, had signed parent/guardians informed consent.

### Sample size and sampling strategies

Based on the previous evidence on the distribution of *S.* *haematobium* and *S.* *mansoni* along the Lake Malawi (Lake Nyasa in Tanzania) basin^23^, village located along the shoreline of the lake were purposively sample based on their proximity to the lake. The WHO recommended sample size and sampling strategies for evaluating prevalence and intensity of infection for the purpose of assessing the need for control measure was used^28,29^. This cross-sectional survey(s) involved a random selection of 150–250 pre-school and school aged children from the list of the registered students in school attendance book on the day of sample collection. The sampling technique used in this study has been described elsewhere^11,30^.

## Data collection

### Collection of participants information

A pre-tested questionnaire was used to collect children’s demographic information (age, sex) and height. The questionnaire also collected information relating to participation in mass drug administration. The questionnaire for children aged 3–6 years was administered to their caregivers (parents/guardians). The data collection form was translated to Kiswahili and back-translated to English before data entry.

### Parasitological screening for *Schistosoma mansoni* using Kato Katz technique

Duplicate Kato Katz (KK) thick smears were prepared from a single stool samples collected from each participating child. A template of 41.7 mg was used to make the thick smears. The prepared smears were examined by two independent laboratory technicians trained on KK technique. For quality assurance, 20% of all the positives and negative KK thick smears were re-examined by a third laboratory technician blinded of the results of the other two technicians.

### Examination of *Schistosoma mansoni* circulating cathodic antigen

A single urine sample was obtained from 574 pre-school and school aged randomly selected children who participated in the study due to limited number of the POINT OF CARE CIRCULATING CATHODIC ANTIGEN TESTS (POC-CCA) test kits. The POC-CCA test active infection by detecting the CCA produced by live worms in the blood vessels and excreted in urine of infected individuals^31^ (http://www.rapid-diagnostics.com/). The test was performed by laboratory technicians trained using the manufacturers manual.

### Parasitological screening for *Schistosoma haematobium* infection

A single urine sample was collected from all pre-school and school aged children participated in the study. Urine collection was done between 10:00 AM and 2:00 PM at the agreed site in the community and at the school environment. Collected urine sample were grossly examined for macro haematuria and using a urine dipstick/urinalysis reagent strips (MISSION, Expert, USA) was used to determine micro-haematuria. A filtration technique was used for screening of urine samples and a light microscopy was used to examined urine filters for presence of *S.* *haematobium* eggs^32^. Each sample was examined independently by two medical laboratory technicians and at the end of each fieldwork day, 20% of all the positive and negative samples were re-examined as described above.

### Geographical distribution of infection

To determine the geographical distribution of the infection prevalence, positions of all the villages/10 primary schools participating in the study were mapped using a Tropical Data Kit Tools using the GPS software installed in a mobile phone or tablets^33^. All the collected coordinates were imported in ARVVIEW Software (https://www.esri.com/en) and allowed the maps of the district to be generated (Figs. 3 and 4).

### Data analysis

Data were double entered in a MICROSFOT EXCEL SHEET, cleaned and exported to STATA VERSION 15 (StataCorp, 2017). The focus of the analysis was to determine the prevalence of *S.* *haematob*ium and *S.* *mansoni* based as diagnosed using Kato Katz technique, Urine filtration technique POINT OF CARE CIRCULATING CATHODIC ANTIGEN TESTS (POC-CCA) rapid test. Continuous (age, eggs intensities) variables were summarised using mean ± standard deviation (SD). Frequencies/proportions/categorical were compared using Chi-square (χ^2^) or Fisher’s exact tests and continuous variables were compared using t-test. For *S.* *mansoni* eggs, arithmetic mean eggs counts were obtained from the counts of four KK smears and multiplied by 24 to obtain the individuals’ eggs per gram of feaces. The mean egg counts for *S.* *mansoni* between sex and age groups were compared using either t-test (two groups) or ANOVA (for more than two groups). Intensity of infection was categorized according to WHO criteria, 1–99 epg, 100–399 epg and ≥ 400 defined as low, moderate and heavy intensities of infection^34^. For *S.* *haematobium* infection, the Geometrical Mean eggs output was estimated based only on infected children because the number of infected children was too small. Infection intensities were classified into two categories as per WHO recommendation^35^ (i) light infection (< 50 eggs/10 ml of urine) and (ii) heavy infections (≤ 50 eggs/10mls of urine).

### Ethics approval and consent to participate

Ethical approval for this study was provided by the National Ethical Committee, The National Institute for Medical Research, Tanzania (cert. NIMR/HQ/R.8a/Vol.1X/3061) and the methods used to collect the presented data followed the recommended standard operating procedures and the study was conducted according to Helsinki recommendation. The study received further permission from regional and districts authorities of Ruvuma and Nyasa district. The village authorities were also informed before data collection was done. Parents and guardians of pre-school children and school aged children received information through the village government communication channels and the school. Written informed assent and consent form were received from children and parents/guardians before participation in the study. An assent form was developed for children aged 9–13 years and they were informed about the study procedures. Confidentiality was maintained throughout the study. All children diagnosed with either *S*. *haematobium* or *S.* *mansoni* were treated with PZQ (40 mg/kg) according to WHO recommendation.

## Results

A total of 1560 pre-school (34.9%) and school aged children (65.1%) aged 1–13 years participated in the study. Of these children, 46.3% (722/1560) and 53.7% 838/1560) were female and male respectively. Table 1 shows distribution of sex and age among the study participants.

**Table 1:** Sex and age distribution among the study participants in selected villages surrounding Lake Nyasa, north-western Tanzania.

Sex	Age groups (years)		
	1–5	6–10	11–13
Female	247 (45.3%)	448 (47.6%)	27 (36.9%)
Male	298 (54.7%)	494 (52.4%)	46 (63%)
Total	545	942	73

### Prevalence of haematuria, *Schistosoma haematobium* and infection intensity

The overall prevalence of haematuria (both macro and micro haematuria) was 0.9% (14/1560) with no sex difference (χ^2^ = 0.6346, *P* = 0.43). Based on the urine filtration technique, 0.83% (13/1560) (95%CI: 0.48–1.4) of the children screened had *S.* *haematobium* infection. Almost 79% (11/13) of the children detected with eggs of *S.* *haematobium* in their urine samples were from the age group 6–10 years. In relation to *S.* *haematobium* infection intensity, 61.5% (8/13) and 38.5% (5/13) of the children had light and heavy infection intensity respectively. The overall geometrical mean egg count was 18.53 mean egg counts/10 ml of urine (95%CI: 5.9–57.6).

### Prevalence and intensity of *Schistosoma mansoni* based on Kato Katz technique

**Figure 1:**
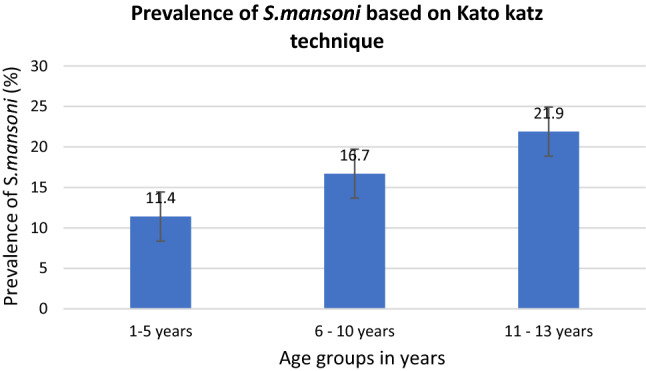
Prevalence of *S.* *mansoni* based on Kato Katz technique among pre-school and school aged children in villages surrounding Lake Nyasa, Southern Tanzania.

The overall prevalence of *Schistosoma* *mansoni* was 15.1% (95%: 13.4–16.9), with no gender difference but with significant difference among age groups (χ^2^ = 10.3642, *P* = 0.001). The age group 6–10 years and 11–13 years had the highest prevalence of *S.* *mansoni* infection at 16.7% and 21.9% respectively (Fig. 1). The overall arithmetic mean eggs intensity of *S.* *mansoni* was 34.7 eggs per gram of feaces (34.7 ± 141.06 epg, 95%CI: 27.7–41.7), with no significant difference between sex (t = 1.1567, *P* = 0.24) but with significance difference between age groups (F = 4.99, *P* = 0.01). The age groups 6–10 years (41.88 ± 168.4 epg) and 11–13 years (53.9 ± 138 epg) had the highest mean eggs intensities. Based on the WHO criteria for infection intensity, 40%, 42.1% and 17.8% of the children had mild, moderate and heavy infection intensities.

### Prevalence of *Schistosoma mansoni* infection based on Point-of-Care Circulating Cathodic antigen rapid test

**Figure 2:**
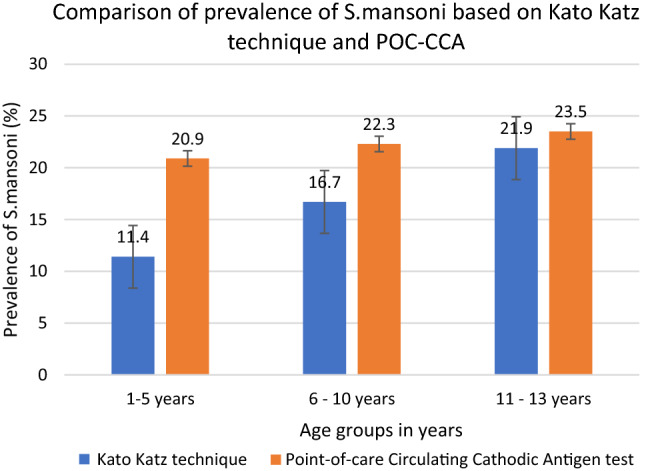
Prevalence of *S.* *mansoni* based on Kato Katz technique and Point-of-Care Circulating Cathodic Antigen rapid test among pre-school and school aged children in villages surrounding Lake Nyasa, Southern Tanzania.

A total of 574 randomly school children from all the selected study villages were screened for *S.* *mansoni* infection using Point-of-Care Circulating Cathodic Antigen test. Of the screened children, 244 (42.5%) were children under the age of five years. The overall prevalence of *S.* *mansoni* based on the POC-CCA test was 21.8% (95%CI: 18.5–25.3) with neither age (χ^2^ = 0.2181, *P* = 0.8) nor sex (χ^2^ = 2.0679, *P* = 0.2). Almost 79% of the children diagnosed with *S.* *mansoni* were detected by both POC-CCA and Kato Katz technique (Fig. 2).

### Distribution of *Schistosoma mansoni* and *Schistosoma haematobium* in the selected villages along Lake Nyasa

**Figure 3:**
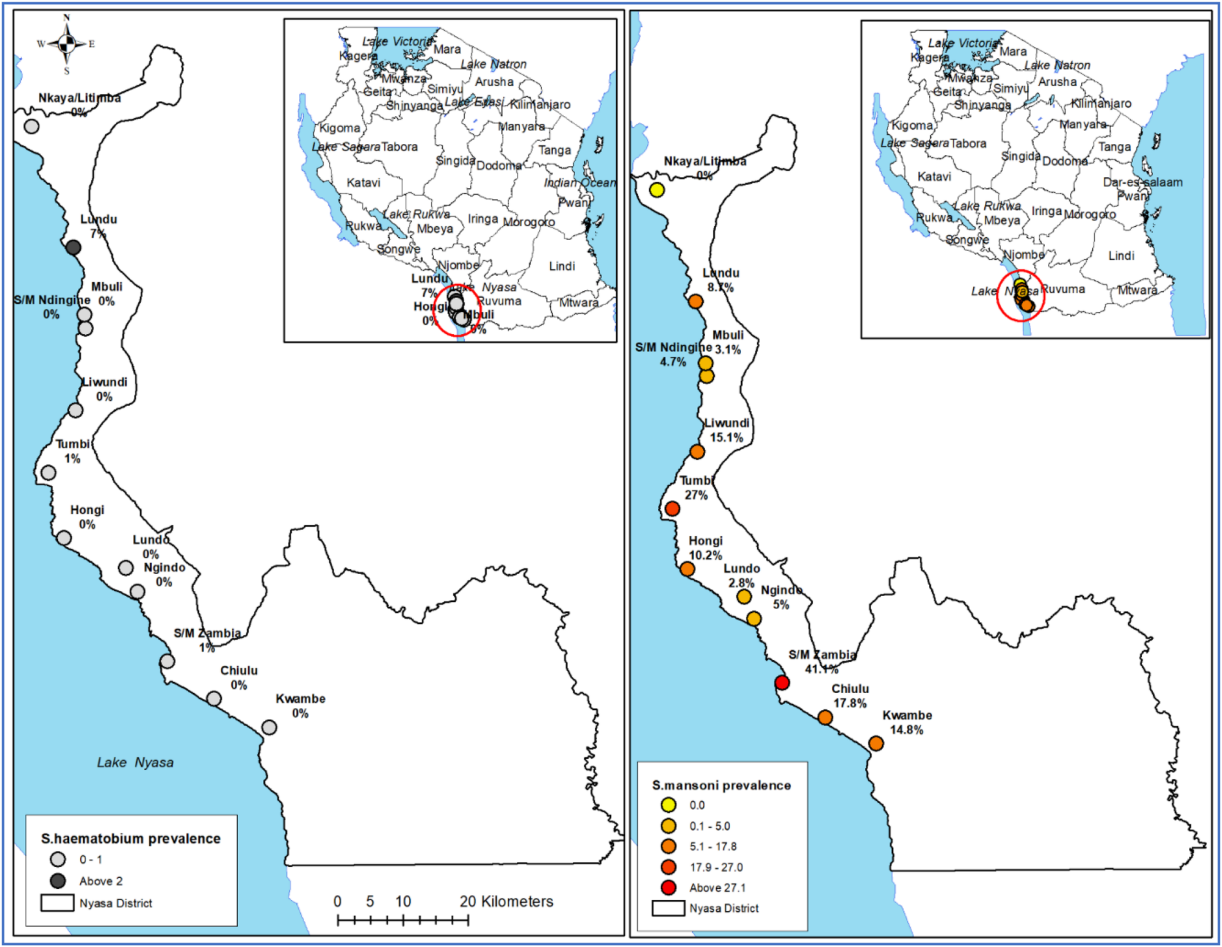
The geographical distribution of *Schistosoma* *mansoni* and *Schistosoma* *haematobium* in selected villages along Lake Nyasa shorelines in southern Tanzania.

**Figure 4:**
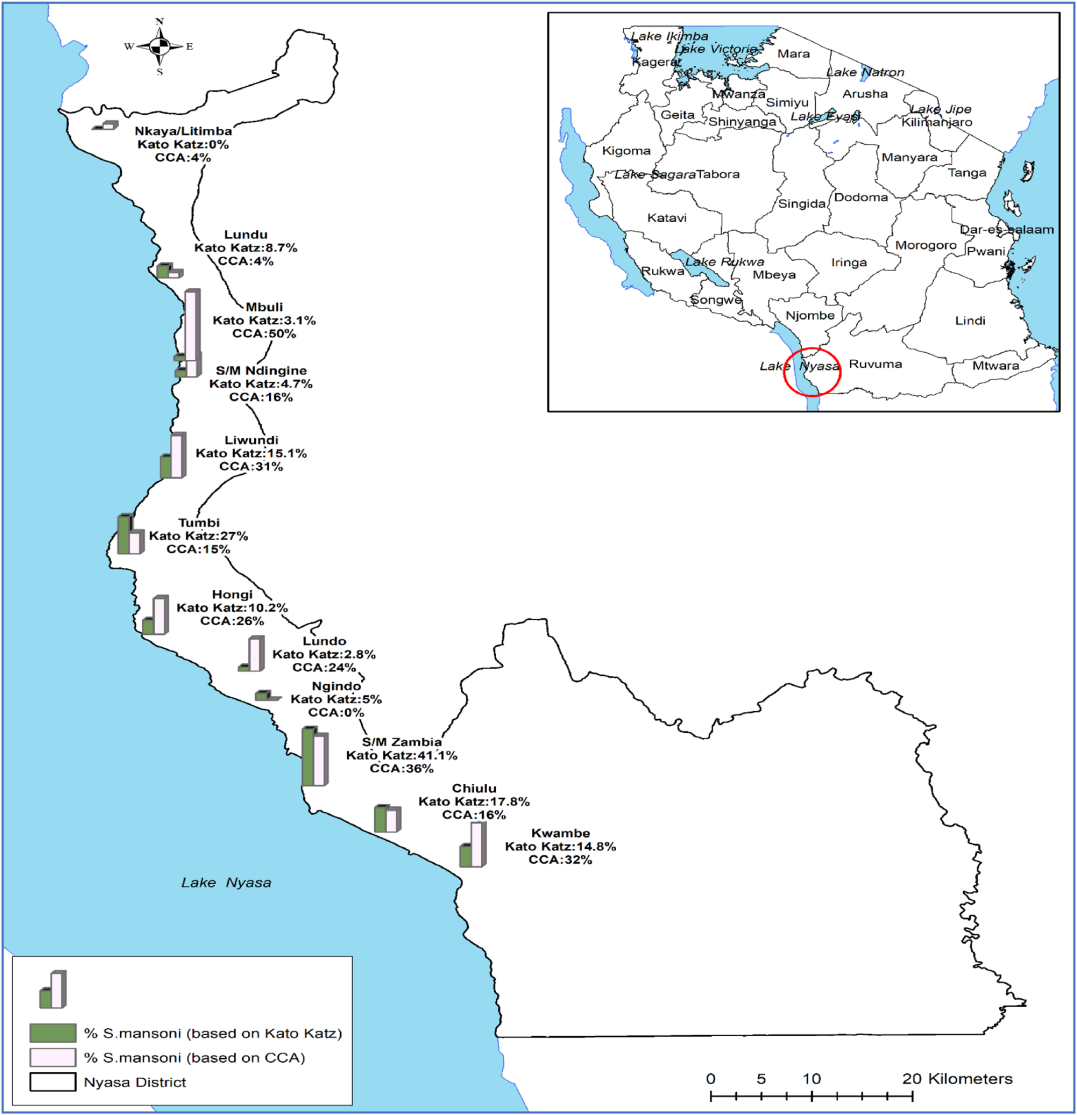
Geographical prevalence of *Schistosoma* *mansoni* based on Kato Katz technique and point-of-care circulating cathodic antigen tests in villages surrounding Lake Nyasa, Southern Tanzania.

Using both KK technique and POC-CCA, Schistosoma mansoni was the most frequently diagnosed in all the ten villages selected for the study. Using the KK technique, the prevalence of *S.* *mansoni* ranged from 2.8 to 41.1% in Zambia (Mbaba-bay area). Villages which were located on the southern end of the Lake Nyasa had significantly higher prevalence of *S.* *mansoni* based on both test than villages which were located on the north side of the lake (χ^2^ = 178.7838, *P* = 0.001) (Figs. 3 and 4).

For urogenital schistosomiasis using urine filtration technique, out of the ten villages included in the study, *S.* *haematobium* was detect in three (3) villages only, which included Lundu (1%), Tumbi (1%) and Zambia-Mbaba-bay (7%) (Fig. 3).

## Discussion

Although Tanzania bears a disproportionately high burden of schistosomiasis in the African region, the mapping of schistosomiasis prevalence remains incomplete in many areas of the country. To our knowledge, this is the first cross-sectional study which provides evidence of existence and endemicity of urogenital and intestinal schistosomiasis in communities living along the shorelines of Lake Nyasa, southern Tanzania. The prevalence of schistosomiasis infection increased with increase in age of the children. There was a variation in prevalence of *S.* *haematobium* and *S.* *mansoni* between villages, with villages located on the southern part of the lake having high prevalence of *S.* *mansoni* infection (diagnosed by POC-CCA and KK technique) compared to village located on the northern part of the lake. The prevalence of *S.* *haematobium* was very low and cases were detected only in three villages located on north and southern part of the lake. Our findings on the existence of *S.* *haematobium* and *S.* *mansoni* infections along the shorelines of Lake Nyasa are consistent with previous research conducted in communities living along shorelines of the Lake Malawi in Malawi^23^.

### Prevalence and intensities of *S. mansoni* and *S. haematobium* infection

The overall prevalence of *S.* *mansoni* based on Kato Katz technique was lower compared to the prevalence of > 50% recorded among pre-school and school aged children along the shoreline of the Lake Victoria in north-western Tanzania^10,26,36^. However, the observed prevalence was higher compared to studies conducted along the same lake shore communities in Malawi where they recorded a prevalence 3.9% based on Kato Katz technique^23^. Using Point of Care Circulating Cathodic Antigen test, the overall prevalence of schistosomiasis was 21.8% which was lower compared to 80%^26^ recorded in pre-school children in Ukerewe island and 71.3%^13^ recorded among school aged children along the Lake Victoria in north-western Tanzania^13^ and on islands of Lake Victoria in Uganda^13,37^. The variation in prevalence of *S.* *mansoni* from one epidemiological setting to another can be explained by the focal distribution of *S.* *mansoni*, abundance and competence of the intermediate snail hosts, proximity of the villages/communities to transmission sites such as the lake, effectiveness of the control intervention such as MDA and level of environmental contamination with human feaces^10–12,25^.

The prevalence and intensity of *S.* *mansoni* increased with increase in the age group of the children, the youngest had lower prevalence and intensity of infection compared to those aged above 6 years. This is a common observed in *S.* *mansoni* endemic communities and the age prevalence curve shows that *S.* *mansoni* prevalence and infection intensities usually peaks at the age groups 6–19 years, thereafter, declines with increased age^38^. However, it is worth noting that in high transmission setting like around the Lake Victoria basin in north-western region of Tanzania, the prevalence and intensities of infection are similar to all age groups^6,39^. The present study noted that in this setting, *S.* *mansoni* infection starts at very young ages (< 5 years), meaning that the age group is exposed at early life and this calls for inclusion of the age group in treatment programme. This observation is consistent to what has been reported in schistosomiasis endemic areas in sub-Saharan Africa^26,40,41^.

In contrast, the prevalence of *S.* *haematobium* in the screened pre-school and school-aged children was lower compared to reports of previous studies conducted in Zanzibar (5.4%)^42^ and north-Western Tanzania (34.8%)^43^. The observed prevalence of *S.* *haematobium* in the present study remained lower than the prevalence of 56.2% to 94% of *S.* *haematobium* observed among schools aged children in lakeshore communities of Lake Malawi^23^ and in the inland communities in Malawi (15.3–57.1%)^23^. The contrasting findings on the *S.* *haematobium* infection prevalence in communities living along the shorelines of the lake between the two countries could partly be explained by the focal distribution of the *S.* *haematobium* intermediate hosts in a defined geographical area, while in Malawi, high transmission appears to occur along the lakeshore communities compared to inland communities^23^, in Tanzanian side of the same lake, *S.haematobium* transmission is very low but the area maintain high transmission of *S.* *mansoni*. Perhaps, transmission of *S.* *haematobium* in the area surrounding Lake Nyasa occurs more on the inland areas compared to lakeshore areas. The present study did not include the inland communities located out of the lake shorelines and malacological surveys were not done. The inclusion of these areas could have given a different picture of *S.* *haematobium* and *S.* *mansoni* transmission levels in the district. This call for a follow-up study.

On the other hand, all children identified to be infected with *S.* *haematobium* were aged > 6 years and majority had low infection intensities. Supporting evidence from previous studies which have reported that children aged > 6 years remains at high risk of *S.* *haematobium* than the youngest age groups^43^. This can be explained by increased duration and frequencies of human-water contact activities among the members of the age group which exposes them to infection^43^. The absence of *S.* *haematobium* infection in children under five in the present study can also be explained by a low transmission of *S.* *haematobium* in the study setting or limitation of parasitological techniques to identify children with low infection intensities, which is common observation in children under-fives^42^. In sub-Saharan Africa, *S.* *haematobium* infection has been noted to start at young age^44–46^, for instance in Zimbabwe, *S.* *haematobium* infection starts at young ages (< 5 years) and these children carries high infection intensities^47^. Probably, high transmission of the parasite occurs in water bodies which are accessible by children under five years and this exposes them to infection^48^. Previous studies noted that distance from household to an open water sources increases the risk of *S.* *haematobium* infection^49^. A recent knowledge indicates that the presence of the actual habitat close to the household(s) was a predictor of *S.* *haematobium* infection rather than distance^43^.

### Variation in prevalence of *S. mansoni* and *S. haematobium* by villages

A noted variation in prevalence of *S.* *haematobium* and *S.* *mansoni* infection prevalence was observed between the villages involved in the study. *Schistosoma* *mansoni* infection was present in all the selected villages at varying prevalence, with the villages located on the southern part of the lake having the highest prevalence compared to villages located on the northern part of the lake. Consistent observation has been reported in *S.* *mansoni* endemic areas, with a variation occurring just within a single village^10–12,25^. In contrast, in lake Malawi, the prevalence of *S.* *mansoni* was very low in villages located along the shorelines^23^. An inverse relationship between proximity to the lake and prevalence of *S.* *mansoni* prevalence and infection intensity has been noted by previous studies^12,25^, with villages located away from the lake shoreline having the lowest prevalence^25^. For *S.* *haematobium*, cases were only identified in only three (3) villages which were located on the northern part of the lake. The prevalence was very low compared to what was observed in villages located along the shoreline of lake Malawi^23^, which indicated that transmission of *S.* *haematobium* was high in villages located along the lake shoreline compared to inland villages^23^. In addition, in Malawi, villages located on the southern part of the lake had higher prevalence of *S.* *haematobium* mainly in shallow water of the lake^23^. Overall, the observed difference in prevalence of *S.* *mansoni* and *S.* *haematobium* between villages indicate that, in these villages, transmission of *S.* *mansoni* is high and occurs along the lake shoreline, while the transmission of *S.* *haematobium* is low along the shoreline’s villages. However, this observation needs follow-up studies to understand in detail the ecology of transmission of *S.* *mansoni* and *S.* *haematobium* infections with a focus to understand the transmission patterns, involved intermediate hosts and their distributions and geographical variations among villages located at different points from the lake shoreline. In lake Malawi, where transmission of *S.* *haematobium* is very high, the main intermediate host is *Bulinus* *nyassanus* and in the inland villages both *Bulinus* *nyassanuss* and *Bulinus* *globosus* are involved^23^. In the area, the transmission of *S.* *haematobium* is limited to areas where the *B.* *nyassannus* occurs, mainly in shallow water close to the shore^23^. For *S.* *mansoni*, the main intermediate host reported was *Biomphalaria* *pfeifferi* and was noted to be predominant in the inland villages compared to lakeshore villages^23^, explaining the low prevalence of the infection in lakeshore communities. However, this information is not available on the Tanzanian side of lake Nyasa and calls for extensive malacological studies in this area.

### Implication for mass drug administration

The prevalence and distribution of *S.* *mansoni* and *S.* *haematobium* infection in the study villages has implication on the planning and implementation of mass drug administration. The WHO health organization recommends MDA against schistosomiasis when the prevalence exceeds 10%^34^. Considering this recommendation, most of the villages requires MDA against *S.* *mansoni* infection at least one round every year with exception of only two villages which will require one rounds of MDA after every one year^34^. For *S.* *haematobium* infection, if resources are enough, a test and treat approach is recommended to reduce the wastage of drugs. The fact that *S.* *mansoni* and *S.* *haematobium* are co-endemic in some villages, MDA against one of them especially *S.* *mansoni* will take care of the *S.* *haematobium* infection. Our study has noted that children aged 5 years and below were also infected by *S.* *mansoni* infection and the age group is normally excluded from MDA programme^44,45^. It is worthwhile to note that children under-fives carry heavy infection intensity similar to primary school aged children and adult individuals in endemic areas^26,40,47^. Available evidence indicated that in endemic areas children under-five bears significant hepatosplenic and urogenital morbidities associated with *S.* *mansoni* and *S.* *haematobium* infection^44,50^. These evidences indicate the need for inclusion of children under-fives for preventive chemotherapy for schistosomiasis and studies have demonstrated that the currently available PZQ drug has less side effects in the age group^47^. Thus, planning and implementation of next MDA rounds should consider inclusion of this age groups.

Although the current study has shown for the first time the geographical distribution of *S.* *haematobium* and *S.* *mansoni* in villages located along the Lake Nyasa in southern Tanzania, it is worthwhile to note that the study included only selected ten (10) villages located along the shorelines. Village located outside the lake shoreline and those located far south of the lake were not included. This may limit the generalizability of the findings to other areas outside the district. In addition, the study did not include ultrasound studies to understand the level of hepatosplenic and urogenital morbidities associated with *S.* *mansoni* and *S.* *haematobium* in pre-school and school aged children. Future studies should cover these gaps.

## Conclusion

Our findings demonstrate that villages in closest proximity to lake Nyasa in southern Tanzania had the highest *S.* *mansoni* prevalence while the prevalence of *S.* *haematobium* was very low (below 10%). These findings provide guide for planning and implementation of mass drug administration against schistosomiasis in Nyasa district. Furthermore, the findings have further demonstrated the need for paediatric praziquantel formulation or any feasible intervention to address schistosomiasis in pre-school children. Lastly, the findings call for further studies to understand the ecology of schistosomiasis transmission (malacological surveys) in villages surrounding lake shore and inland areas of Nyasa district.

## Data Availability

Data supporting the write-up and conclusion of this article are provided with the articles. Raw data are available for sharing upon reasonable request through the institutional review board/ethical review committee.

## Abbreviations

PPF: Periportal fibrosis
MDA: Mass drug administration
SAC: School aged children
PSAC: Pre-school aged children
PZQ: Praziquantel
KK: Kato Katz technique
POC-CCA: Point-of-care circulating cathodic antigen
GPS: Geographical positioning system
NIMR: National Institute for Medical Research
NTD: Neglected tropical diseases
